# Comparisons of Soybean and Wheat; in the Focus on the Nutritional Aspects and Acute Appetite Sensation

**DOI:** 10.3390/foods11030389

**Published:** 2022-01-29

**Authors:** Akihiro Maeta, Masahiro Katsukawa, Yaeko Hayase, Kyoko Takahashi

**Affiliations:** 1 Department of Food Science and Nutrition, School of Food Science and Nutrition, Mukogawa Women’s University, 6-46, Ikebiraki, Nishinomiya 663-8558, Japan; maeta_a@mukogawa-u.ac.jp; 2 Product Development Division, Kikkoman Food Products Company, 338, Noda 278-0037, Japan; mkatsukawa@mail.kikkoman.co.jp (M.K.); yhayase@mail.kikkoman.co.jp (Y.H.)

**Keywords:** gluten-free, soybean, satiety, appetite, visual analog scale (VAS)

## Abstract

Soybean flour is often used as a gluten-free ingredient. We aimed to compare the nutrients and the difference in satiety of soybean and wheat after ingestion. We measured the amounts of polyphenol and oxygen radical absorbance capacity (ORAC) and examined the acute appetite sensation after the ingestion of soybean powder and bread powder. Japanese women were enrolled in the meal tests. Participants were provided with 18 g of bread or soybean powder, 180 g of yogurt, and 285 mL of bottled water. Subjective satiety (hunger, appetite, satiety, and stomach fullness) was measured using a visual analog scale 120 min after sample ingestion. The polyphenol content and ORAC were 2- and 12-folds higher, respectively, in soybean powder than in bread flour. In the meal tests, the area under the curve (AUC) of satiety 60–120 min after ingestion (*n* = 44) was significantly higher for soybean powder than bread powder. The AUCs of hunger and appetite 60–120 min after ingestion were significantly lower for soybean powder than bread powder. The effect sizes of hunger and appetite by soybean powder were 0.341 and 0.424, respectively. Thus, these results suggest that soybean is a healthy food and soybeans maintain satiety and suppress hunger more than bread flour.

## 1. Introduction

Wheat is one of three largest consumed grains in the world and is a raw material for various foods such as bread, pasta, and cakes. Gluten is one of the main proteins found in wheat and plays an important role in the manufacture of bread, pasta, udon (Japanese noodles), etc. However, gluten is also a large cause of wheat allergy (WA), celiac disease (CD), and non-celiac gluten sensitivity (NCGS). WA is characterized by the production of wheat protein immunoglobulin E (IgE) antibodies and the development of symptoms of immediate-type food allergies [1]. In Japan, wheat is the third-most common causative food [1]. CD is an enteropathy with autoimmune characteristics that is triggered by gluten-containing foods in susceptible individuals [2]. CD is found mainly in Caucasians, occurring in 1 per 130–300 individuals in Western European [2]. NCGS is defined as “a syndrome characterized by intestinal and extra-intestinal symptoms related to the ingestion of gluten-containing food, in subjects that are not affected by either CD or WA” [3]. NCGS prevalence rates range from 0.49% to 14.9% in the Western Europe adult population [3]. Therefore, the development of gluten-free products is essential to treat these diseases.

Cereal foods such as rice powder and corn starch are used as a gluten-free ingredient. However, a high intake of rice increases the risk of type 2 diabetes in Japanese women [4]. Carbohydrates are the main nutrient of cereal foods such as wheat, rice, and corn. Moreover, a low-carbohydrate, high-fat, and protein diet can decrease the risk of type 2 diabetes in Japanese women [5]. Low-carbohydrate and ketogenic diets have been noted as key factors for weight-loss. Johnstone et al. reported that a high-protein, ketogenic diet (P:F:C = 30:66:4) reduced body weight compared to the control diet (P:F:C = 30:34:36) [6]. Moreover, slightly increasing protein intake for several months by 0.1 g/kg/d in a dose-dependent manner over a range of doses from 0.5 to 3.5 g/kg/d may increase or maintain lean body mass [7]. Therefore, the utilization of non-cereal foods as wheat alternatives is important, and legumes such as soybeans are good materials.

Globally, soybeans are mostly used as feedstock for soybean oil [8]. In Japan, approximately 30% of soybeans are edible, which is higher than that in other regions [9]. Soybean is also used as a raw material for tofu, miso, and soy sauce production in Japan. Moreover, kinako is a roasted soybean powder used for the preparation of Japanese sweets. Soybeans are rich in proteins. In comparison, 100 g of bread flour contains 11.8 g of protein, while 100 g of soybean contains 33.8 g of protein [10]. Moreover, the amino acid score of soybeans is 100, whereas that of bread flour is approximately 49 [10]. Soybeans score approximately 16 on the glycemic index (GI), whereas that of bread scores approximately 75 [11]. Moreover, the functionality of soybeans has attracted considerable attention. A prospective cohort study in Japan reported that a higher intake of fermented soy was associated with a lower risk of mortality [12]. Harland et al. reported that modest soybean protein intake (around 25 g) in adults with normal or mild hypercholesterolemia induces significant reductions in total and low-density lipoprotein (LDL) cholesterol [13]. Anderson et al. reported in a meta-analysis that soy protein, rather than animal protein, significantly decreased the serum concentrations of total cholesterol, LDL cholesterol, and triglycerides [14]. Additionally, it was reported that peptides derived from soy protein inhibit the activities of angiotensin-converting enzyme [15] and promote the expression of the LDL receptor [16]. Moreover, β-conglycinin, which is the main storage protein in soybean protein and accounts for 30% of protein, has some bioactive functions. Peptides derived from β-conglycinin inhibit fatty acid synthase [17]. Dietary intake of soybean β-conglycinin suppressed body weight gain by increasing fibroblast growth factor (FGF-21) gene expression [18,19]. Furthermore, Hirotsuka revealed that soybean β-conglycinin is a food ingredient that decreases serum triglyceride and reduces visceral fat by ingesting 2.3 g/d [20]. Furthermore, soybeans are high in isoflavones that act like phytoestrogens (plant estrogens). Thus, soybeans are useful for maintaining human health.

There are many reports regarding the manufacture of gluten-free bread, including soybean flour [21,22]. Ribotta et al. reported that heated soybean flour showed better results, yielding a well-aerated crumb structure and a higher bread volume than non-heated soybean flour [23]. Moreover, the optimal soybean flour level was 125 and 150 g/kg [23]. Taghdir et al. reported that the total score of the sensory evaluation was better in bread samples containing 15% soybean flour than in 5% and 10% soybean flour [24]. Soybean β-conglycinin has potential as a structuring agent in gluten-free breads [25]. Additionally, it was found that roasted soy flour containing 1% hydroxypropyl-methylcellulose decreased the hardness of bread and reduced the “beany flavor” of whole soy bread [26].

However, few studies have investigated the nutritional and physiological differences after ingesting bread and soybean flour in the focus on a gluten-free ingredient. Thus, we focused on two points: first, the nutrient composition of soybean powder and bread flour, and second, satiety maintenance, and hunger suppressed after the ingestion of soybean powder and bread powder.

## 2. Material and Methods

### 2.1. Nutrients, Total Polyphenol Contents, and Antioxidant Ability of Soybean and Bread Flour

The nutrient compositions of soybean and bread flour were obtained from the Standard Tables of Food Composition in Japan 2020 [10].

The total polyphenol content and antioxidant ability were measured using the Folin-Ciocalteu reagent method and oxygen radical absorbance capacity (ORAC) method, respectively. We used soybean powder (Puffmin F–puffed full-fat soybean powder), Kikkoman Food Products Company, Noda, Japan) and bread flour (Camellia, Nisshin Seifun Group Inc., Tokyo, Japan).

### 2.2. Preparation of Soybean and Bread Powders for Meal Tests

We used bread powder (natural panko, Frystar Co., Ltd., Kanagawa, Japan) because raw wheat flour can cause diarrhea and food intoxication [27]. We used soybean powder (Puffmin F). Approximately 18 g of each powder was packed in an aluminum bag at the Kikkoman Food Products Company (Noda, Japan). The energies of bread and soybean powders per 100 g were 356 and 450 kcal, containing 12.5 and 38.8 g protein, 2.2 and 23.2 g fat, and 71.6 and 27.1 g carbohydrate (4.0 and 11.3 g fiber), respectively. The powder was mixed with yogurt (Meiji Bulgaria Yogurt, Meiji Co., Ltd., Tokyo, Japan; Table 1). The energy of the yogurt per a production (180 g) was 144 kcal, containing 6.2 g protein, 5.5 g fat, and 17.5 g carbohydrate (<0 g fiber).

### 2.3. Study Subjects

This study was approved by the Ethics Committee of Mukogawa Women’s University (Permit Number: 19-105) and Kikkoman General Hospital (Permit Number: KC-RD22). The purpose and protocol of this study were explained to all subjects, and written informed consent was obtained from all participants. We recruited healthy female Japanese students over 20 years of age. The exclusion criteria were as follows: subjects who were diagnosed with an allergy to wheat, soybean, and milk; obese subjects; subjects with abnormal blood pressure or hematological abnormalities; and subjects who had digestive, circulatory, or metabolic diseases in the previous year. Before satiety experiments, height and body composition were measured using InBody 270 (InBody Japan Inc., Tokyo, Japan), and the participants answered a brief self-administered diet history questionnaire (BDHQ) [28].

In designing the number of study participants, the significance level was set to 5% and the power (1-β) was set to 95%, considering type I and type II errors. With reference to the report by Cohen [29], the effect size was set to 0.5, and the statistically required sample size was calculated to be 47.

### 2.4. Experimental Design and Assessment of Subjective Satiety and Hunger Using a Visual Analog Scale

**Figure 1:**
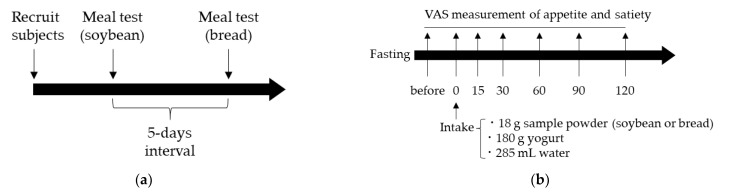
Experimental schedule (**a**) and follow chart of the meal test (**b**).

The experimental schedule is illustrated in Figure 1a. This study was performed at the Department of Food Science and Nutrition, Mukogawa Women’s University (Hyogo, Japan) in February 2020. Meal tests were separated by 5 days (washout for 5 days). The meal tests schedule is illustrated in Figure 1b. On the days of the experiments, the participants arrived at the university at 09:20 a.m. Fifteen minutes after arrival, participants recorded their first visual analog scale (VAS) score for subjective satiety and appetite in the fasting state (baseline). Then, the participants were provided with bread or soybean powders, 180 g of yogurt, and 285 mL of bottled water (I LOHAS, Coca-Cola (Japan) Co., Ltd., Tokyo, Japan). Participants were instructed to ingest the entirety of the powder-mixed yogurt and water over 10 min (mins). Subsequently, participants recorded the VAS scores for subjective satiety and appetite (time = 0). Subjective satiety and appetite were assessed at 15, 30, 60, 90, and 120 min after meal consumption.

We modified the Japanese-translated VAS score for subjective satiety and appetite analysis [30]. The VAS score was configured by feelings of hunger, appetite, satiety, and stomach fullness, as follows:

1. “How hungry do you feel?”. Responses could range from ”I do not feel hungry at all” to ”I feel extremely hungry”.
2. “How does your appetite feel?”. Responses could range from “I do not feel appetite at all” to ”I feel extremely appetite”.
3. “How satiety do you feel?”. Responses could range from ”I do not feel satiety at all” to “I feel extreme satiety”.
4. “How much stomach fullness do you feel?”. Responses could range from ”I do not feel full at all” to “I feel extremely full”.

The area under the curve (AUC, min × mm) was calculated from 0 to 60 min and 60 to 120 min.

### 2.5. Statistical Analysis

Values are presented as a median (25–75th percentile) value. The effect size index (d) was calculated using Cohen [29] and Suzukawa [31] methods. G power version 3.1.9.2 (Statistical Power Analyses (Heinrich Heine University), Dusseldorf, Germany) [32,33] was used to design the number of study participants.

$$d=\left|\;\frac{{\bar{X}}_{Soybean-Bread}}{{\bar{\sigma }}_{Soybean-Bread}}\times \sqrt{n\;}\right|\times \sqrt{\frac{1}{n}}$$

${\bar{X}}_{Soybean-Bread}\;\mathrm{is}\;\mathrm{the}\;\mathrm{mean}\;\mathrm{of}\;\mathrm{differece}\;\mathrm{between}\;\mathrm{soybean}\;\mathrm{and}\;\mathrm{bread}\;\mathrm{powder}$.

${\bar{\sigma }}_{Soybean-Bread}\;\mathrm{is}\;\mathrm{the}\;\mathrm{standard}\;\mathrm{error}\;\mathrm{of}\;\mathrm{differece}\;\mathrm{between}\;\mathrm{soybean}\;\mathrm{and}\;\mathrm{bread}\;\mathrm{powder}$.

n is sample numbers.

$d\;\mathrm{is}\;\mathrm{an}\;\mathrm{effect}\;\mathrm{size}\;\left(\mathrm{small}:\;d=0.20,\;\mathrm{medium}:d=0.50,\;\mathrm{large}:d=0.80\right).$

Differences at each time points were analyzed using the Wilcoxon signed-rank test (non-parametric paired t test). Differences were considered significant (*p* < 0.05). Prism by GraphPad (version 5.0; GraphPad Software, San Diego, CA, USA) was used for all of the analyses.

## 3. Results

### 3.1. Nutrient Compositions of Soybean and Bread Flour

Most of the nutrients, except for carbohydrates, were more abundant in soybean than in bread flour (Table 2). The potassium (K), calcium (Ca), and magnesium (Mg) contents of soybean were 21.3-, 10.6-, and 9.6-fold higher, respectively, than those of bread flour (Table 2). Vitamin B_1_ (VB_1_), vitamin B_2_ (VB_2_), vitamin B_6_ (VB_6_), folate, and biotin of soybean were 7.9, 6.5, 8.5, 16.3, and 16.2-fold higher, respectively, than those of bread flour (Table 2).

### 3.2. Total Polyphenol Contents and Antioxidant Ability of Soybean and Bread Flour

The polyphenol content of soybean powder was approximately 2-fold higher than bread flour (Table 3). Moreover, the antioxidant ability was approximately 12-fold stronger in soybean powder than the bread flour (Table 3).

### 3.3. Subject Information

Of the enrolled subjects, 54 completed satiety experiments for both bread and soybean powders, while 10 were excluded due to incomplete or inadequate VAS answers. Finally, data from 44 participants were used for the analysis (Table 4).

### 3.4. Subjective Satiety, Stomach Fullness Hunger and Appetite after Bread or Soybean Powder Intake

**Figure 2:**
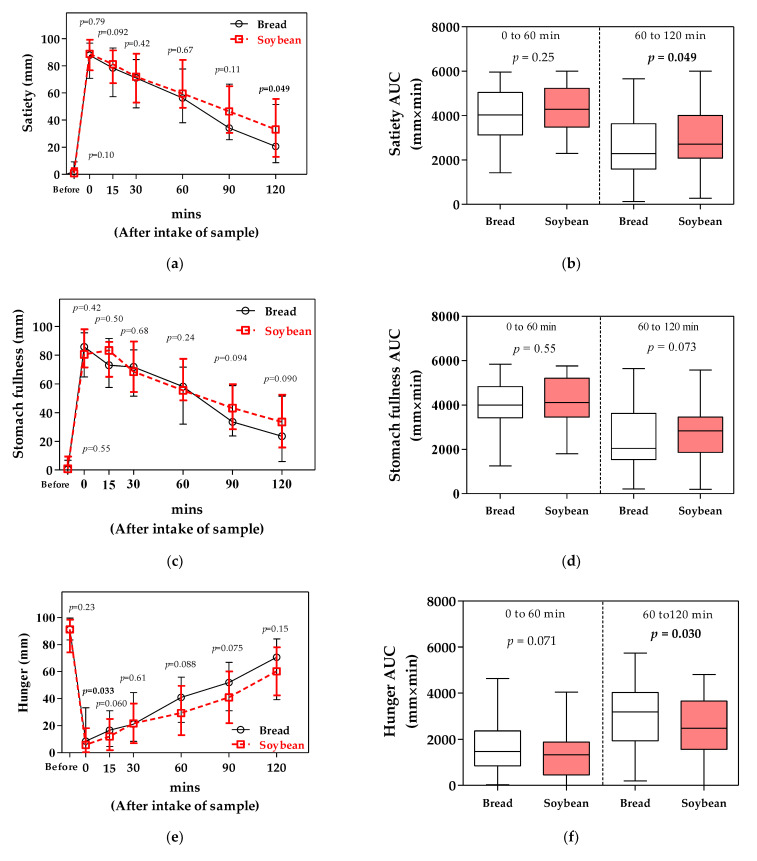

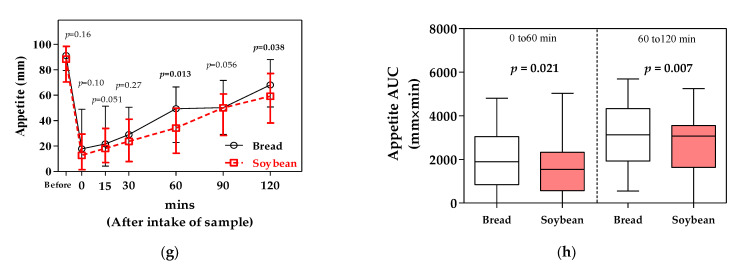
VAS score and AUC of satiety (**a**,**b**), stomach fullness (**c**,**d**), hunger (**e**,**f**), and appetite (**g**,**h**) after bread (black circle) and soybean (red square) powder ingestion.

The satiety VAS score 120 min after ingestion was significantly higher for soybean powder than for bread powder (Figure 2a). Moreover, the AUC for satiety 60 to 120 min after ingestion was significantly higher for soybean powder than for bread powder (Figure 2b). However, in the stomach fullness VAS score, there were no significant difference between before ingestion and 120 min after (Figure 2c). Furthermore, the AUCs for stomach fullness at 0 to 60 and 60 to 120 min after ingestion were not significantly different between the bread and soybean powder (Figure 2d).

There was no significant difference in the hunger VAS score between bread and soybean powder ingestion after 15–120 min (Figure 2e). However, the AUCs for hunger 60 to 120 min after ingestion were significantly lower for soybean powder than for bread powder (Figure 2f). The appetite VAS scores 60 and 120 min after ingestion were lower for soybean powder than for bread powder (Figure 2g). Moreover, AUCs for appetite after ingestion were significantly lower for soybean powder than for bread powder at both intervals of 0 to 60 and 60 to 120 min (Figure 2h).

### 3.5. Effect Size of the Maintenance of Satiety and the Suppression of Hunger and Appetite by the Intake of Soybean Powder

Effect size is the degree of difference in the mean between groups and the strength of the association between some factors. However, many satiety studies have not calculated effect sizes. Cohen defined a small effect size as approximately 0.2, a medium effect size as approximately 0.5, and a large effect size as approximately 0.8 [29].

The effect size of satiety by intake of soybean powder was 0.274, meaning that it was a small effect (Table 5). The effect size of hunger and appetite by intake of soybean powder was 0.341 and 0.424, respectively, measuring between the small and medium effects (Table 5).

## 4. Discussion

In this study, we focused on two points: first, the nutrient composition of soybean and bread flour, and second, satiety maintenance and hunger suppression after ingesting soybean powder and bread powder.

Soybean is a low carbohydrate and high protein gluten-free ingredient. Moreover, soybean contains more minerals and vitamins than bread flour. It has been reported that the dietary Na/K ratio is associated with hypertension prevalence in Japanese individuals [34]. Furthermore, Mg intake is associated with the prevalence of prefrailty and frailty in older Japanese women [35]. Miyake et al. reported that a low intake of folate, VB_6_, and VB_2_ during pregnancy increased the risk of low prosocial behavior, childhood hyperactivity problems, low prosocial behavior, and childhood emotional problems in five-year-old Japanese children [36]. Soybean contains polyphenols, such as genistein and daidzein, and has a high antioxidant ability. The dietary intake of antioxidant foods is associated with low mortality from cardiovascular, heart, and cerebrovascular diseases [37]. Moreover, soybean isoflavones act like phytoestrogens (plant estrogens). Thus, soybean is a healthy gluten-free ingredient. In particular, soybeans can contribute to maintaining and improving the health of women.

Satiety was maintained after ingesting soybean and bread flour. In this study, the median BMI of the participants was 20.8. In the 2018 Japan National Health and Nutrition Survey, the mean ± standard error of the BMIs of Japanese women aged 20 to 29 years old was reported as 21.0 ± 3.1, respectively [38]. Moreover, the median intake of the amount of energy (kcal), protein (g), fat (g), carbohydrate (g), total dietary fiber (g), and NaCl (g) for Japanese women aged 20 to 29 years old were reported as 1631, 60.3, 54.3, 208.7, 10.5, and 8.4, respectively [38]. In this study, the median intake of these nutrients was 1619, 61.30, 50.95, 213.0, 10.85, and 8.55, respectively. Therefore, we hypothesize that the body composition and nutritional intake of the participants in this study were comparable to those of Japanese women of the same generation.

The intake of nutrients is related to the sustention of satiety and the suppression of hunger and appetite. In 1955, Mayer proposed glucostatic and lipostatic hypotheses [39]. In terms of the sugar content, 100 g of bread flour contains 69.0 g sugar while 100 g of soybean contains 8.0 g sugar [10]. The GI of soybean is approximately 16, whereas the GI of bread is approximately 75 [11]. High GI foods induced the early and sharp change of blood glucose than low GI foods [40]. Niwano et al. indicated that the relatively early and sharp decline to below the baseline in blood glucose was a key to the earlier disappearance of satiety and the earlier return of appetite and hunger [40]. In this experiment, the intake of soybean-maintained satiety and suppressed hunger and appetite compared to that for bread. Rigamonti et al. reported that the intake of whey proteins promotes satiety and suppresses hunger compared to maltodextrin intake [41]. It has been reported that a high-protein diet suppresses postprandial hunger compared to a high-carbohydrate diet [6,42]. Dietary protein intake may influence appetite sensations by enhancing fullness or satiety [43,44,45]. Moreover, some characteristic proteins sustain satiety and suppress hunger or appetite compared to other proteins. Hursel et al. reported that the α-lactalbumin-enriched high-protein yogurt drink suppresses hunger and the desire to eat more than the whey-enriched high-protein yogurt drink [46]. Nishi et al. reported that soybean β-conglycinin peptone suppresses the amount of food intake compared with water and wheat gluten peptone in a rat model [47]. Hira et al. reported that soybean β-conglycinin hydrolysate was effective in enhancing fullness and reducing hunger sensations in healthy humans [48]. Therefore, we hypothesize that the satiety and suppression of hunger can be maintained by the ingestion of soybean-related compounds, such as soybean β-conglycinin, and the increased intake of proteins.

Physical (such as mastication and abdominal bloating) and physiological stimulation (such as increased glycemia and secretion of hormones, e.g., leptin, glucagon-like peptide-1(GLP-1), cholecystokinin (CCK)), and peptide YY (PYY)) are involved in satiety [49,50]. In this experiment, the feeling of stomach fullness, which shows physical stimulations, was not significantly different between the bread and soybean powder. The viscosity of soybean powder with yogurt was lower than those of bread powder with yogurt (data not shown). Thus, we considered that the satiety difference between soybean- and wheat-related physiological stimulations was low. Rigamonti et al. reported that the plasma levels of GLP-1 and PYY after the whey protein intake were higher than those after maltodextrin intake [41]. Veldhorst et al. concluded that GLP-1 release evoked by a high-protein meal is relatively higher than that evoked by a high carbohydrate intake, and PYY release is stimulated by a high-protein meal [45]. Nishi et al. reported that CCK levels in the portal plasma of rats that ingested soybean β-conglycinin peptone were approximately 5-fold higher than those in rats that ingested water [47]. Moreover, Serrano et al. reported that insulin secretion after the ingestion of 500 mL soymilk (20 g carbohydrate, of which 13.5 g was sugar) was 1.8-fold higher than that in the control beverage (20.5 g glucose) [51]. Insulin also promotes leptin secretion by mast cells. Leptin suppresses feeding behavior [52]. However, in this experiment, we did not measure objective indexes such as leptin, GLP-1, and CCK. It will be necessary to elucidate the detailed mechanism of postprandial satiety sustention and hunger suppression in soybeans from the changes in physiological stimulation.

There are limitations of this study. First, all the subjects were young Japanese women, and satiety was only measured via subjective VAS scores. Future studies should carry out a meal test to target men and other generations, and measure objective indexes such as GLP-1 and CCK. Second, differences in the macronutrients of soybean powder and bread powder might relate the differences of subjective satiety and appetite after sample ingestions. However, we considered that it is one of the characteristics of soybeans that it has more nutrients such as protein, lipids, vitamins, and minerals than those of wheat, and that even if the results of this study are caused by differences in macronutrients, the benefits can be obtained by using soybean powder instead of wheat flour. Third, we did not ensure that the participants were not giving into social desirability bias for this experiment. Fourth, we did not control for some other factors such as eating behavior or dietary patterns. In this study, differences within the same individual were evaluated. Thus, we consider that the effects of individual differences in dietary behavior or pattern are low for the results of subjective satiety and appetite after sample ingestion.

## 5. Conclusions

Soybean flour is often used as a gluten-free ingredient. We focused on two points: first, the nutrient composition of soybean powder and bread flour, and second, satiety maintenance and hunger suppression after ingesting soybean powder and bread powder. We considered soybean to be more useful than bread flour for the prevention of lifestyle diseases. Moreover, soybean powder has potential for postprandial satiety sustention and hunger suppression compared to bread powder. We expect that soybean is useful as a gluten-free ingredient in terms of nutrient composition and physiological effects.

## Figures and Tables

**Table 1:** Nutrition information of ingesting samples ^1^.

Item	Soybean Powder (18 g) with Yogurt (180 g)	Bread Powder (18 g) with Yogurt (180 g)
Weight, g	198	198
Energy ^2^, kcal	225	207
Protein, g	13.2	8.5
Fat, g	9.7	5.9
Carbohydrate, g	22.4	30.4
Of which fiber, g	4.1	1.4

^1^ Macronutrients were calculated from the nutrients label of each products. ^2^ Energy was calculated by Atwater factors. Energy (kcal) = protein (g) × 4 (kcal/g) + fat (g) × 9 (kcal/g) + (carbohydrate (g) − fiber (g)) × 4 (kcal/g) + fiber (g) × 2 (kcal/g).

**Table 2:** Nutrient compositions of soybean and bread flour ^1^.

Items	Soybean	Bread Flour
Food code ^1^	04023	01020
Energy, kcal/100 g	422	365
Protein, g/100 g	33.8	11.8
Amino acid score	100	49
Fat, g/100 g	19.7	1.5
Carbohydrate, g/100 g	29.5	71.7
Total dietary fiber, g/100 g	21.5	2.7
Water soluble, g/100 g	6.1	1.2
Insoluble, g/100 g	15.4	1.5
Mineral		
Na, mg/100 g	1	Tr
K, mg/100 g	1900	89
Ca, mg/100 g	180	17
Mg, mg/100 g	220	23
P, mg/100 g	490	64
Fe, mg/100 g	6.8	0.9
Zn, mg/100 g	3.1	0.8
Cu, mg/100 g	1.07	0.15
Mn, mg/100 g	2.27	0.32
I, μg/100 g	0	0
Se, μg/100 g	5	39
Cr, μg/100 g	3	1
Mo, μg/100 g	350	26
Vitamin (V)		
VA, μgRAE/100 g	1	(0)
VD, μg/100 g	(0)	0
VE (α- Tocopherol), mg/100 g	2.3	0.3
VK, μg/100 g	18	(0)
VB_1_, mg/100 g	0.71	0.09
VB_2_, mg/100 g	0.26	0.04
Niacin, mgNE/100 g	10.2	3.1
VB_6_, mg/100 g	0.51	0.06
VB_12_, μg/100 g	(0)	0
Folate, μg/100 g	260	16
Pantothenic acid, μg/100 g	1.36	0.77
Biotin, μg/100 g	27.5	1.7
VC, mg/100 g	3	(0)

^1^ Nutrients were quoted from the Standard Tables of Food Composition in Japan 2020 [10]. NE—niacin equivalents; RAE—retinol activity equivalents; V—vitamin.

**Table 3:** Total polyphenol contents and antioxidant ability of soybean and bread flour.

Items	Soybean Powder	Bread Flour
Total polyphenol content ^1^, g/100 g of powder	0.31	0.16
Antioxidant ability (Total ORAC), μM TE/g of powder	99	8

^1^ Equivalent amount of catechin. Abbreviations: ORAC—oxygen radical absorbance capacity.

**Table 4:** Body composition and nutrition intake of the participants ^1^.

Items	*n* = 44
Age, year	21 (21–21)
Body composition	
Height, cm	158.2 (154.8–161.6)
Weight, kg	51.55 (47.83–57.48)
BMI, kg/m^2^	20.80 (19.23–21.95)
Body muscle, kg	20.55 (19.15–21.40)
Body fat, %	27.55 (24.30–31.03)
BDHQ	
Energy, kcal	1619 (1414–1919)
Protein, g	61.30 (51.03–73.28)
Fat, g	50.95 (44.95–63.08)
Carbohydrate, g	213.0 (183.3–243.8)
Total dietary fiber, g	10.85 (8.25–12.63)
NaCl, g	8.55 (7.13–10.0)

^1^ Values are presented as median (25–75th percentile). BMI—body mass index; BDHQ—brief self-administered diet history questionnaire; NaCl—sodium chloride.

**Table 5:** Effect size of the intake of soybean powder at 60 to 120 min.

Items	Difference of AUC at 60 to 120 min ^1^ (Soybean–Bread)	Effect Size (d) ^2^
Satiety	343 ± 1268	0.274
Hunger	−391 ± 1147	0.341
Appetite	−435 ± 1025	0.424

^1^ Values are presented as mean ± standard error (*n* = 44). ^2^ The effect size index (d) was calculated using the Cohen [29] and Suzukawa [31] methods. AUC—area under curve; min—minute.
